# Inguinal Dermoid Cyst Associated With Ipsilateral Undescended Testis in an Adolescent Male: A Case Report

**DOI:** 10.1002/ccr3.72219

**Published:** 2026-03-09

**Authors:** Muhammad Hassaan Javaid, Muddassir Khalid, Muhammad Sohaib, Fred Segawa

**Affiliations:** ^1^ Department of Surgery Shifa College of Medicine Islamabad Pakistan; ^2^ Department of Surgery Nishtar Medical University Multan Pakistan; ^3^ Department of Surgery Gomal Medical College Peshawar Pakistan; ^4^ College of Health Sciences Makerere University Kampala Uganda

**Keywords:** adolescent male, case report, dermoid cyst, undescended testis

## Abstract

Groin swellings are the most frequent presenting clinical signs, which most frequently arise due to an inguinal hernia or lymphadenopathy. There are also rare congenital lesions, such as dermoid cysts, which can present similarly to common lesions, also leading to a diagnostic challenge. Inguinal dermoid cysts, particularly those that present with an undescended testis, are extremely rare, and there are sparse reported cases. An 18‐year‐old male patient presented with painful, slowly increasing swelling in the left inguinal canal for 3 months. On physical examination, a firm, non‐reducible, tender mass and empty left hemiscrotum were noted. Ultrasonogram revealed a heterogeneous hypoechoic mass containing echogenic foci for hair or calcification, and no presence of the left testis within the scrotum. Tumor markers were within normal limits. At opening, inguinal exploration, a well‐defined cystic mass with thick sebaceous material and hair was excised, and orchiopexy of the viable undescended testis was performed. Histopathology showed a dermoid cyst with stratified squamous epithelium, sebaceous glands, and hair follicles. The postoperative course was uneventful with no recurrence on 4‐month follow‐up. Inguinal dermoid cysts are rare developmental cysts that are also seen as lipomas or hernias. Their association with undescended testes makes them even more challenging to diagnose. Ultrasonography is the preferred imaging before surgery, and surgical resection is the preferred management to prevent recurrence. This case illustrates the importance of dermoid cysts in the differential diagnosis of inguinal masses, particularly in this patient with cryptorchidism, and highlights the importance of surgical exploration and histopathological diagnosis in management.

## Key Clinical Message

This case highlights the importance of considering dermoid cysts in inguinal swellings, especially with undescended testes. Early surgical exploration and histopathological confirmation are crucial for accurate diagnosis, complete excision, and recurrence prevention. Awareness can enhance timely diagnosis and improve outcomes for similar rare presentations.

## 1 Introduction

Groin lumps represent a usual clinical dilemma encountered across several medical specialties, including primary care, emergency medicine, and surgical disciplines. While the massive majority of cases are attributed to the inguinal hernias (75%–80%) and lymphadenopathies (15%–20%), a wide spectrum of differential diagnosis must be considered to avoid misdiagnosis [1, 2]. Possible differentials include pre‐peritoneal lipoma, supernumerary pectineus bursa, internal oblique hematoma, varicoceles, undescended testis, a variety of adenopathies, and atypical cystic structures [2].

Dermoid cysts or mature teratomas are congenital lesions that contain mature tissues from at least two germ layers. They can be cutaneous or subcutaneous. They most frequently occur in locations such as the face, neck, or scalp. Less commonly, they are encountered intracranially, intraspinally, or peri‐spinally, along the anterior abdominal wall, or in other unusual sites. Dermoid cysts as a cause of inguinal canal lumps are quite rare [1, 3]. Dermoid cysts in the inguinal canal, along with the spermatic cord, are exceedingly rare.

To the best of our knowledge, very few cases of inguinal dermoid cysts coexisting with an ipsilateral undescended testis have been reported in adolescents, making this presentation exceptionally rare. According to a literature review, only 12 cases of dermoid spermatic cord have been reported properly in the literature so far [4].

We therefore report a case of an 18‐year‐old adolescent male with a 3‐month history of painful and progressively enlarging swelling in the left inguinal region. This case is rare not only because of its very rare occurrence but also because of its overlapping symptoms mimicking a lipoma or a hernia and the presence of an undescended left Testis, which makes it a diagnostic as well as a management challenge.

‘This case report has been reported in line with the SCARE checklist [Kerwan A, Al‐Jabir A, Mathew G, Sohrabi C, Rashid R, Franchi T, Nicola M, Agha M, Agha RA. Revised Surgical CAse REport (SCARE) guideline: An update for the age of Artificial Intelligence. Premier Journal of Science 2025:10;100,079] [5].

## 2 Case History

An 18‐year‐old male presented with a 3‐month history of a painful, progressively enlarging swelling in the left inguinal region. The swelling did not improve with analgesics. On inspection, there was an oval, non‐reducible, non‐pulsatile swelling in the left inguinal region, without overlying skin changes. On palpation, the mass was firm, tender, and lacked a cough impulse. Examination of the scrotum revealed an empty left hemiscrotum, while the right testis was normal. The patient was born at home without standard early‐life checks on genital development. Despite multiple observations by relatives and the person himself, the absence of one testicle went unaddressed medically due to a lack of symptoms. It was only when discomfort emerged in the groin area that concern arose. Medical records verifying two testes after delivery or in younger years do not exist. Confirmation of normal anatomy during infancy cannot be established through written evidence.

### 2.1 Differential Diagnosis

- Inguinal hernia (reducible, incarcerated, or strangulated)
- Spermatic cord lipoma.
- Inguinal lymphadenopathy
- Undescended testis presenting as an inguinal mass.
- Epidermoid (epidermal inclusion) cyst
- Dermoid cyst (mature cystic teratoma)
- Spermatic cord cyst.
- Hydrocele of the spermatic cord.
- Varicocele
- Hematoma of the inguinal canal.
- Psoas abscess tracking into the groin.
- Inguinal canal soft tissue tumor (e.g., liposarcoma, leiomyoma)
- Testicular or paratesticular neoplasm
- Femoral hernia (less likely but considered)

This case posed a diagnostic challenge due to overlapping clinical features with more common inguinal pathologies, particularly inguinal hernia and spermatic cord lipoma, underscoring the importance of maintaining a broad differential diagnosis.

### 2.2 Investigations

**FIGURE 1:**
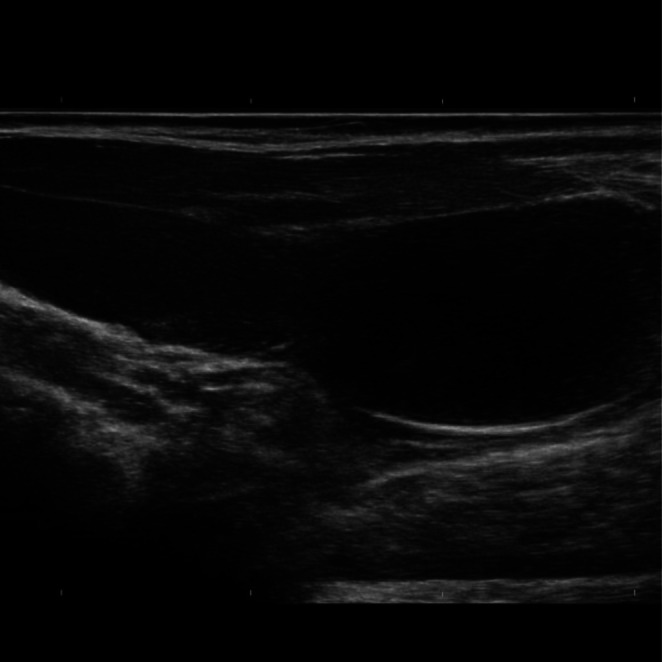
Ultrasonography of the left inguinal region. The left testis was not visualized within the scrotum.

Routine laboratory investigations, including complete blood count, liver function tests, and renal function tests, were normal. Tumor markers (AFP, β‐hCG, and LDH) were within normal limits. Scrotal ultrasonography revealed a hypoechoic, heterogeneous mass measuring 3.8 × 4.8 cm in the left inguinal canal. Fluid collection of about 6 mL was noted in the left inguinal region. The lesion contained echogenic foci suggestive of hair or calcification. The left testis was not visualized in the scrotum, and no hernial sac or bowel contents were detected (Figure 1).

Microscopic examination showed a clearly defined area, bordered by stratified squamous tissue showing abundant granular cells. Within its walls sat fully developed skin structures—sebaceous and sweat glands, along with intact hair roots packed with dead protein material. Inside the cavity lay organized layers of keratin intertwined with strands of hair. Absent entirely were signs of early development stages, abnormal nuclei, cell division, or cancerous change. Notably missing also were any traces of testis tissue, duct‐like tubules, hormone‐producing cells, or reproductive cell types, ruling out gonadal involvement. This pattern supports the diagnosis of a benign dermoid cyst.

### 2.3 Treatment

The patient underwent open inguinal exploration under general anesthesia.

**FIGURE 2:**
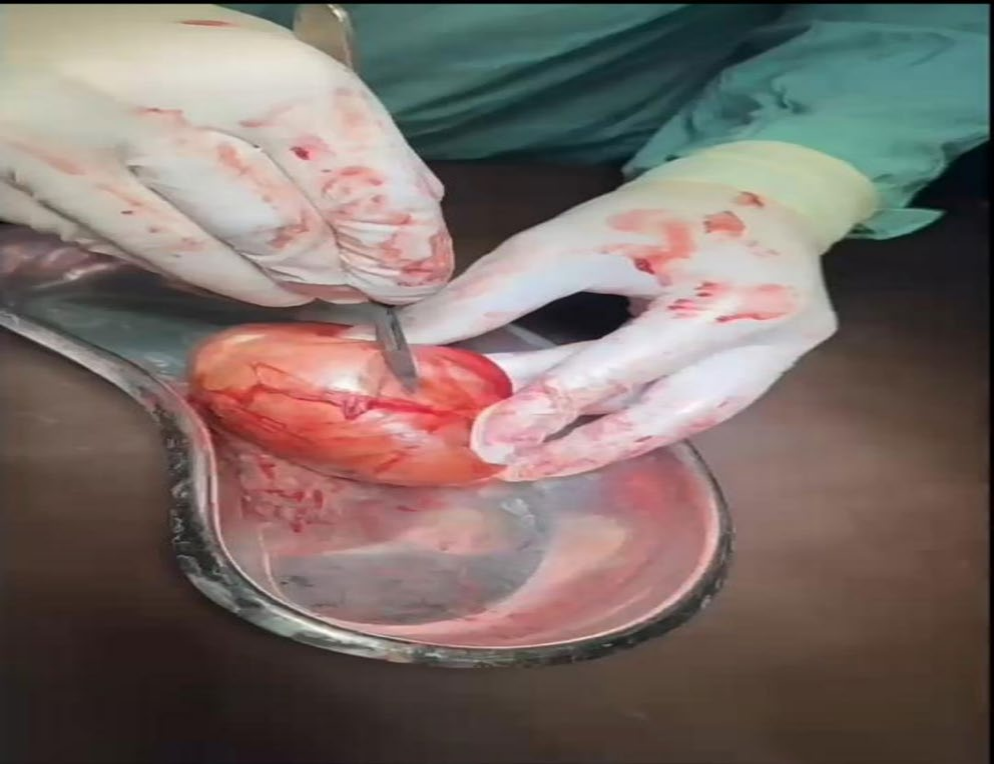
Excised dermoid cyst (and opening the cyst for gross examination).

A well‐circumscribed cystic lesion was identified along the spermatic cord, separate from any hernial sac. On incision, the cyst contained thick, malodorous white paste interspersed with hair, consistent with a dermoid cyst. The undescended left testis was located proximal to the cyst and was viable. The cyst was completely excised (Figure 2), followed by posterior wall repair using 2–0 polypropylene. Orchiopexy was performed in the same setting.

Gross examination showed a cyst filled with keratinous debris and hair. Microscopic analysis confirmed a stratified squamous epithelial lining with sebaceous and sweat glands, as well as hair follicles. No features of malignancy or immature elements were identified. A small fluid‐filled sac appeared during surgery, surrounded by a clear border and attached to the tissue running alongside the left testicle. Though found nearby, this growth sat apart from the testis, which rested higher up in the spermatic cord. Separation was evident—the cyst did not merge with the testicular structure at any point. Connection remained intact between the epididymis and the ductus deferens, untouched by the lesion. Tissue analysis confirmed no shared framework linking the cyst directly to functional testicular cells.

### 2.4 Outcomes and Follow‐Up

The patient received intravenous ceftriaxone and metronidazole postoperatively. Sutures were removed, and complete wound healing was achieved. At the 4‐month follow‐up, no recurrence was noted. The left testis remained in the scrotum with preserved function. Despite years of undescended testes, the individual learned about a higher chance of developing testicular cancer over time. To monitor health, yearly physical checks were set up, along with ultrasound scans of the scrotum. Awareness became part of care—each person was guided on how to examine their own tissue regularly. Blood tests for tumor signs may return should symptoms or findings suggest the need.

## 3 Discussion

Dermoid cysts primarily result from congenital or acquired developmental anomalies during embryonic fusion [6, 7]. The two primary mechanisms have been proposed: One is displaced pluripotent cells during testicular descent at week 7–12 of gestation, and the second is implantation of epidermal elements due to minor trauma or surgical procedures. Due to their rarity and overlapping symptoms with other common groin pathologies, they are repeatedly misinterpreted as a hernial sac containing omentum, irreducible or incarcerated hernias, or lipomas stemming from the spermatic cord [8].

In fact, up to 60% of inguinal dermoid cysts are initially misdiagnosed as hernias, as exemplified by a case report of Ba‐Shammakh et al., who described a case of a 15‐year‐old male with a dermoid cyst located inside the inguinal hernia sac, [8]. Ultrasonography remains the first‐line imaging modality with 58% sensitivity and 99% specificity in diagnoses of all cases reported of dermoid cyst [9]. Key features of these cysts include a hyperechoic mass with hyperechoic foci (hair, calcifications).

MRI, as a gold standard for complicated or ambiguous cases, shows that fat‐suppressed sequences confirm sebaceous content, and diffusion‐weighted imaging (DWI) helps to exclude malignancy. As our case was not very complicated, there was no need for an MRI. Histologically, dermoid cysts are characterized by highly differentiated squamous epithelial outer lining and underlying fibrous connective tissue, which contains abundant hair follicles, hairs, blood vessels, sebaceous, eccrine, and apocrine glands [1]. Dermoid cyst can be easily distinguished from benign cystic teratomas that usually occur in ovarian, testicular, retroperitoneal, and sacrococcygeal regions by histological and gross examination. In our case, a testicular origin of the dermoid cyst was considered. However, this was deemed unlikely due to the complete anatomical separation of the cyst from the testicular parenchyma intraoperatively and the absence of testicular tissue within the excised specimen on histopathology. Furthermore, the preserved morphology of the testis with intact spermatic cord structures supports an extratesticular origin, consistent with a dermoid cyst of the inguinal canal rather than a testicular teratoma.

Surgical considerations and newer techniques allow a surgeon to use a laparoscopic or open approach, as laparoscopy is gaining traction for bilateral exploration and pediatric cases. Surgical excision must be complete to avoid recurrence, as incomplete resection has been linked to recurrence rates that are up to 15% stated in some studies. Recurrence rate is less than 5% with complete excision. Dermoid cysts in females are predominantly ovarian, but in males, they can be found occasionally in the inguinal region, as was the case with our patient [10]. Orchiopexy is quite necessary as 70% of cases with undescended testes require simultaneous orchiopexy [1]. Laparoscopic techniques are now gaining attention, especially in bilateral or pediatric cases; this procedure offers fast recovery and less post‐operative pain [11].

Although dermoid cysts are benign, there are complications such as infections in up to 5% and spontaneous rupture, which chances are 2%, leading to chemical peritonitis and intra‐abdominal cysts, including torsion and hemorrhage, particularly in intra‐abdominal presentations. Malignant transformation of the cysts is exceedingly rare and is less than 1% [12], but while in other locations—like ovaries or the other intra‐abdominal sites—malignant transformation has been described in dermoid cysts [8, 13].

Several reports from published literature show the diagnostic challenges associated with inguinal dermoid cysts. A case report by Brightmore showed an inguinal dermoid cyst that closely mimics a strangulated hernia [13]. A case reported by Ashraf et al. also documented a dermoid cyst of the inguinal canal, which also emphasized awareness among surgeons [14].

The prognosis for the inguinal dermoid cysts is excellent following complete surgical excision, as the frequency is rare when the cyst is excised intact with the capsule. In the case reported by Leeming et al., no recurrence or postoperative complications were reported after the excision, and this crucially supports the potential of the surgery [2]. Similarly, Ba Shammakh et al. also documented an uneventful postoperative course with no crucial evidence of recurrence at follow‐up [8].

Our case emphasizes the importance of keeping up a high index of suspicion when appraising groin masses, more importantly in males of younger ages with concurrent cryptorchidism, as stated in the recent literature [11]. Preoperative imaging is all‐important, with ultrasound recommended as the first‐line modality due to its high specificity (90%) in detecting dermoid cysts [15]. However, MRI is progressively advised in complicated and ambiguous cases, more specifically when malignant transformation is suspected [16].

In short, clinicians should consider dermoid cysts in the differential diagnosis of masses in the inguinal regions, especially in male patients of young ages with cryptorchidism, make use of MRI in unclear cases, perform complete excision with orchiopexy when indicated, and ensure follow‐up for a long time to maximize outcomes.

## 4 Conclusion

Inguinal Dermoid Cysts are exceedingly rare entities and become more challenging when they are associated with Ipsilateral Undescended Testis and often imitate more usual conditions such as incarcerated hernias or lipomas. Despite the extreme predominance of inguinal hernias, several other pathologic entities may be encountered in the inguinal region. The case highlights that these rare conditions should be taken into consideration while evaluating inguinal swellings, especially with a history of cryptorchidism. The management of inguinal dermoid cysts remains a diagnostic and therapeutic challenge due to their rarity and nonspecific clinical presentation; if encountered preoperatively or intraoperatively, complete surgical excision, which is the ultimate treatment, should be done.

## Author Contributions

**Muhammad Hassaan Javaid:** conceptualization, methodology, resources, writing – original draft. **Muddassir Khalid:** project administration, supervision, writing – review and editing. **Muhammad Sohaib:** investigation, methodology, visualization, writing – original draft. **Fred Segawa:** project administration, validation.

## Funding

The authors have nothing to report.

## Ethics Statement

The authors have nothing to report.

## Consent

Written informed consent was obtained from the patient for publication of this case report and accompanying images. A copy of the written consent is available for review by the Editor‐in‐Chief of this journal on request.

## Conflicts of Interest

The authors declare no conflicts of interest.

## Data Availability

Data can be made available on the reasonable request from corresponding author.

## References

[1] S. S. Basra , V. Verma , R. N. Hiremath , and M. Mittal , “Complicated Inguinal Hernia or Inguinal Dermoid: A Diagnostic Dilemma,” Asian Journal of Pharmaceutical and Clinical Research 16, no. 4 (2023): 1–2.

[2] R. Leeming , M. Olsen , and J. L. Ponsky , “Inguinal Dermoid Cyst Presenting as an Incarcerated Inguinal Hernia,” Journal of Pediatric Surgery 27, no. 1 (1992): 117–118. 1552432 10.1016/0022-3468(92)90125-q

[3] R. M. Engel , R. C. Elkins , and B. D. Fletcher , “Retroperitoneal Teratoma. Review of the Literature and Presentation of an Unusual Case,” Cancer 22, no. 5 (1968): 1068–1073. 5686638 10.1002/1097-0142(196811)22:5<1068::aid-cncr2820220525>3.0.co;2-3

[4] A. Hooshyari , D. Scholtz , F. V. Ordones , and L. P. Vermeulen , “The Scrotal Lump That You Can't Get Above: A Case Report of a Spermatic Cord Dermoid Cyst Mimicking an Incarcerated Inguinal Hernia,” Urol. Case Rep. [Internet] 50 (2023): 102465. 37416753 10.1016/j.eucr.2023.102465 PMC10320485

[5] A. Kerwan , A. Al‐Jabir , G. Mathew , et al., “Revised Surgical CAse REport (SCARE) Guideline: An Update for the Age of Artificial Intelligence,” Premier Journal of Science 10 (2025): 100079.

[6] P. H. Papanayotou and J. G. Kayavis , “Epidermoid Implantation Cyst of the Lower Lip: Report of Case,” J Oral Surg Am Dent Assoc 1965 35, no. 7 (1977): 585–586. 267176

[7] W. L. Epstein , “Epithelial Cysts in Buried Human Skin,” Archives of Dermatology 76, no. 4 (1957): 437–442. 13457427 10.1001/archderm.1957.01550220045009

[8] S. A. Ba‐Shammakh , M. Almaletti , and B. A. Aqel , “Beyond the Norm: Unveiling a Dermoid Cyst in an Inguinal Hernia Case,” Cureus 15, no. 8 (2023): e43736. 37727175 10.7759/cureus.43736 PMC10505832

[9] T. Ergun and H. Lakadamyali , “A Dermoid Cyst of the Round Ligament Clinically Misdiagnosed as Incarcerated Inguinal Hernia,” Acta Chirurgica Belgica 110, no. 1 (2010): 80–82. 20306916 10.1080/00015458.2010.11680571

[10] N. S. Salemis , G. Karagkiouzis , D. Sambaziotis , and E. Tsiambas , “Large Dermoid Cyst of the Spermatic Cord Presenting as an Incarcerated Hernia: A Rare Presentation and Literature Review,” Hernia 14, no. 3 (2010): 321–323. 19669696 10.1007/s10029-009-0542-x

[11] M. Kumar and M. Luthra , “A Rare Association of Giant Congenital Melanocytic Nevus (Bathing Trunk Nevus) With Cryptorchidism and Inguinal Hernia: Case Report,” Journal of Clinical Images and Medical Case Reports 2, no. 6 (2021): 13 accessed, https://jcimcr.org/articles/JCIMCR‐v2‐1470.html.

[12] R. M. Engel , R. C. Elkins , and B. D. Fletcher , “Retroperitoneal Teratoma. Review of the Literature and Presentation of an Unusual Case,” Cancer 22, no. 5 (1968): 1068–1073. 5686638 10.1002/1097-0142(196811)22:5<1068::aid-cncr2820220525>3.0.co;2-3

[13] T. Brightmore , “Dermoid Cyst of the Inguinal Canal Simulating a Strangulated Inguinal Hernia,” British Journal of Clinical Practice 25, no. 4 (1971): 191. 5575917

[14] S. M. Ashraf , B. A. Ansari , N. Iqbal , and A. Ansari , “Dermoid Cyst of the Inguinal Canal,” Journal of the Indian Medical Association 83, no. 4 (1985): 125–126. 4078331

[15] T. Ergun and H. Lakadamyali , “A Dermoid Cyst of the Round Ligament Clinically Misdiagnosed as Incarcerated Inguinal Hernia,” Acta Chirurgica Belgica 110, no. 1 (2010): 80–82. 20306916 10.1080/00015458.2010.11680571

[16] E. K. Outwater , E. S. Siegelman , and J. L. Hunt , “Ovarian Teratomas: Tumor Types and Imaging Characteristics,” Radiographics 21, no. 2 (2001): 475–490, 10.1148/radiographics.21.2.g01mr09475. 11259710

